# High Voltage Flexible Sodium‐Ion Battery Cathode Materials Based on 1D Covalent Organic Framework

**DOI:** 10.1002/advs.202505311

**Published:** 2025-06-23

**Authors:** Shuai Liu, Puiki Leung, Yong Zuo, Meng Sun, Lei Wei, Frank C Walsh, Tianshou Zhao, Qiang Liao

**Affiliations:** ^1^ Chongqing Univ, Key Lab Low Grade Energy Utilizat Technol & Syst MOE Chongqing 400030 China; ^2^ A National Innovation Center for Industry‐Education Integration of Energy Storage Technology School of Energy and Power Engineering Chongqing 400044 China; ^3^ Southern Univ Sci & Technol Dept Mech & Energy Engn Shenzhen 518055 China; ^4^ Electrochemical Engineering Laboratory, Department of Mechanical Engineering, Faculty of Engineering and Physical Sciences University of Southampton Southampton SO17 1BJ UK

**Keywords:** bipolar material, covalent organic frameworks, organic cathode material, sodium ion battery

## Abstract

Covalent organic frameworks (COFs) have emerged as promising electrode materials for sodium‐ion batteries (SIBs) due to their well‐ordered porous structures that facilitate ion storage and transport. However, conventional 2D and 3D COFs often require post‐processing, such as ball milling or carbon compositing, to enhance electrochemical performance. In this study, a 1D imine‐linked COF, *N*,*N*,*N*′,*N*′‐Tetrakis(4‐aminophenyl)‐1,4‐phenylenediamine‐2,6‐pyridinedicarboxaldehyde (TP‐PDA), is synthesized via a one‐step Schiff base reaction, achieving a fully conjugated and porous structure that enables efficient sodium‐ion transport. TP‐PDA is insoluble in organic electrolytes, ensuring stable cycling performance. The material exhibits a high average discharge potential of 3.1 V and delivers a discharge capacity of 124 mAh g^−1^ at 3 A g^−1^ after 1800 cycles, with a capacity retention exceeding 90%. In a full‐cell configuration with a hard carbon anode, the battery maintains a stable capacity of 122 mAh g^−1^ after 10 000 cycles at 1 A g^−1^ without noticeable capacity degradation. Furthermore, the flexible pouch cell retains its electrochemical integrity under bending conditions, demonstrating its potential for flexible and wearable energy storage applications.

## Graphical abstract

This paper designs and synthesizes a novel 1D COF named TP‐PDA, with a high discharge potential (≈3.1 V). Its unique 1D architecture and synergistic bipolar redox centers promote efficient active‐site utilization and charge compensation, while extended π‐conjugation and high crystallinity improve electronic conductivity and facilitate effective charge delocalization.

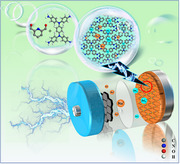

## 1 Introduction

Lithium‐ion batteries (LIBs) have been the prevailing energy storage technology for applications requiring high energy density and long cycle life.^[^ ^1^, ^2^ ^]^ However, the increasing demand for lithium, coupled with its limited global reserves, rising costs, and geopolitical supply risks, has intensified efforts to develop alternative battery chemistries. Sodium‐ion batteries (SIBs) have gained considerable interest as a viable alternative due to the natural abundance of sodium, which is over 1000 times more prevalent than lithium in the Earth's crust. This abundance translates to lower raw material costs and improved scalability for applications, such as grid‐scale energy storage.^[^ ^3^, ^4^, ^5^, ^6^, ^7^ ^]^ Additionally, sodium and lithium exhibit similar electrochemical properties, including comparable redox potentials relative to the standard hydrogen electrode (SHE) (Li vs SHE: ‒3.04 V, Na vs SHE: ‒2.71 V),^[^ ^8^ ^]^ enabling the adaptation of well‐established LIB design principles to SIB development.

Despite these advantages, the widespread adoption of SIBs is hindered by the sluggish diffusion kinetics of Na^+^, primarily due to its larger ionic radius (1.02 Å vs 0.76 Å for Li^+^).^[^ ^9^ ^]^ This increased ionic size restricts transport pathways in conventional inorganic cathode materials, resulting in slow charge‒discharge kinetics and inferior rate performance. Various layered oxide materials, such as NaCoO_2_ and NaNi_1/3_Co_1/3_Mn_1/3_O_2_, have been explored as cathode candidates.^[^ ^10^, ^11^, ^12^ ^]^ However, their practical implementation is hindered by structural instability, phase transitions and limited sodium‐ion mobility, all of which severely restrict long‐term cycling performance.^[^ ^13^, ^14^, ^15^, ^16^ ^]^ In order to deal with this problem, organic electrode materials have emerged as a promising alternative, offering intrinsic advantages, such as structural tunability, renewability and rapid redox kinetics.^[^ ^17^, ^18^, ^19^, ^20^, ^21^ ^]^ The molecular versatility of organic systems enables precise tuning of key battery performance metrics, such as capacity, voltage and cycling stability. However, conventional small organic molecules are prone to dissolution in electrolytes, leading to rapid capacity fading.^[^ ^22^ ^]^ Moreover, poor electronic conductivity further restricts their electrochemical performance.^[^ ^23^, ^24^, ^25^ ^]^

Polymer‐based organic materials offer an effective strategy for addressing these challenges by increasing molecular weight, reducing solubility and extending conjugation to improve charge transport.^[^ ^26^, ^27^, ^28^, ^29^, ^30^ ^]^ Among these, covalent organic frameworks (COFs) have attracted growing interest due to their well‐defined porous structures, chemical stability and tunable redox‐active sites.^[^ ^31^, ^32^, ^33^ ^]^ Their ordered architecture facilitates efficient sodium‐ion transport, making them promising candidates for high‐performance SIB cathodes.^[^ ^34^ ^]^ However, most reported COFs are constructed from rigid aromatic frameworks, which lead to densely packed layered structures, limiting ion accessibility and ultimately compromising both capacity and rate performance.^[^ ^35^ ^]^ Various strategies, such as ball milling and carbon compositing, have been employed to optimize electrochemical properties,^[^ ^36^, ^37^, ^38^ ^]^ but these approaches introduce additional processing complexity, limiting scalability. Furthermore, conventional COFs predominantly feature n‐type redox‐active groups (such as C═O and C═N), which typically result in low discharge voltages (≈1.5–2.5 V *vs* Na^+^/Na), restricting overall energy density.^[^ ^39^ ^]^ Although p‐type COFs exhibit higher discharge voltages, they often suffer from lower theoretical capacities, further constraining their practical utility.^[^ ^40^ ^]^

The design of COF‐based cathode materials that simultaneously deliver high voltage, high capacity and rapid ion transport while maintaining structural stability remains a key research priority.^[^ ^41^ ^]^ While existing COFs primarily utilize either n‐type or p‐type redox centers, achieving a balanced bipolar redox mechanism could enhance charge storage efficiency and improve electrochemical performance.^[^ ^42^ ^]^ In addition, the rigid nature of conventional COFs has resulted in limited mechanical adaptability, preventing their integration into emerging flexible energy storage applications. Thus, the development of COF cathodes with stable, high‐voltage redox mechanisms, improved charge transport and mechanical flexibility is crucial for advancing next‐generation sodium‐ion batteries.^[^ ^43^ ^]^

In this study, a 1D imine‐linked COF, TP‐PDA, was synthesized via a one‐step Schiff base reaction using *N*,*N*,*N*′,*N*′‐Tetrakis(4‐aminophenyl)‐1,4‐phenylenediamine (TP‐NH_2_) and 2,6‐pyridinedicarboxaldehyde (PDA). Unlike conventional 2D COFs, the 1D framework provides enhanced accessibility to redox‐active sites and improved Na^+^ diffusion pathways, facilitating superior electrochemical performance. The charge storage mechanism in TP‐PDA operates via a dual‐ion process, involving both p‐type and n‐type redox centers, which contribute to a high discharge voltage and enhanced sodium storage capacity.^[^ ^44^ ^]^ The synergistic combination of sp^3^‐hybridized nitrogen (sp^3^ N) and C═N redox‐active sites promotes efficient charge compensation, leading to high utilization of active sites.^[^ ^45^ ^]^ Additionally, the extended conjugation and crystallinity of TP‐PDA improve electronic conductivity and charge delocalization, mitigating common limitations in organic electrode materials.^[^ ^46^, ^47^ ^]^ Computational simulations, combined with electrochemical characterizations, demonstrate that TP‐PDA offers both high energy storage capability and mechanical flexibility, achieving a stable discharge capacity of 122 mAh g^−1^ after 10 000 cycles at 1 A g^−1^ with an average discharge voltage of 3.1 V in the TP‐PDA||Hard carbon (HC) full‐battery. It also retains electrochemical integrity under bending conditions, making it a promising candidate for next‐generation sodium‐ion batteries, particularly in flexible and wearable applications.

## 2 Results and Discussion

### 2.1 Synthesis and Characterization

**Figure 1:**
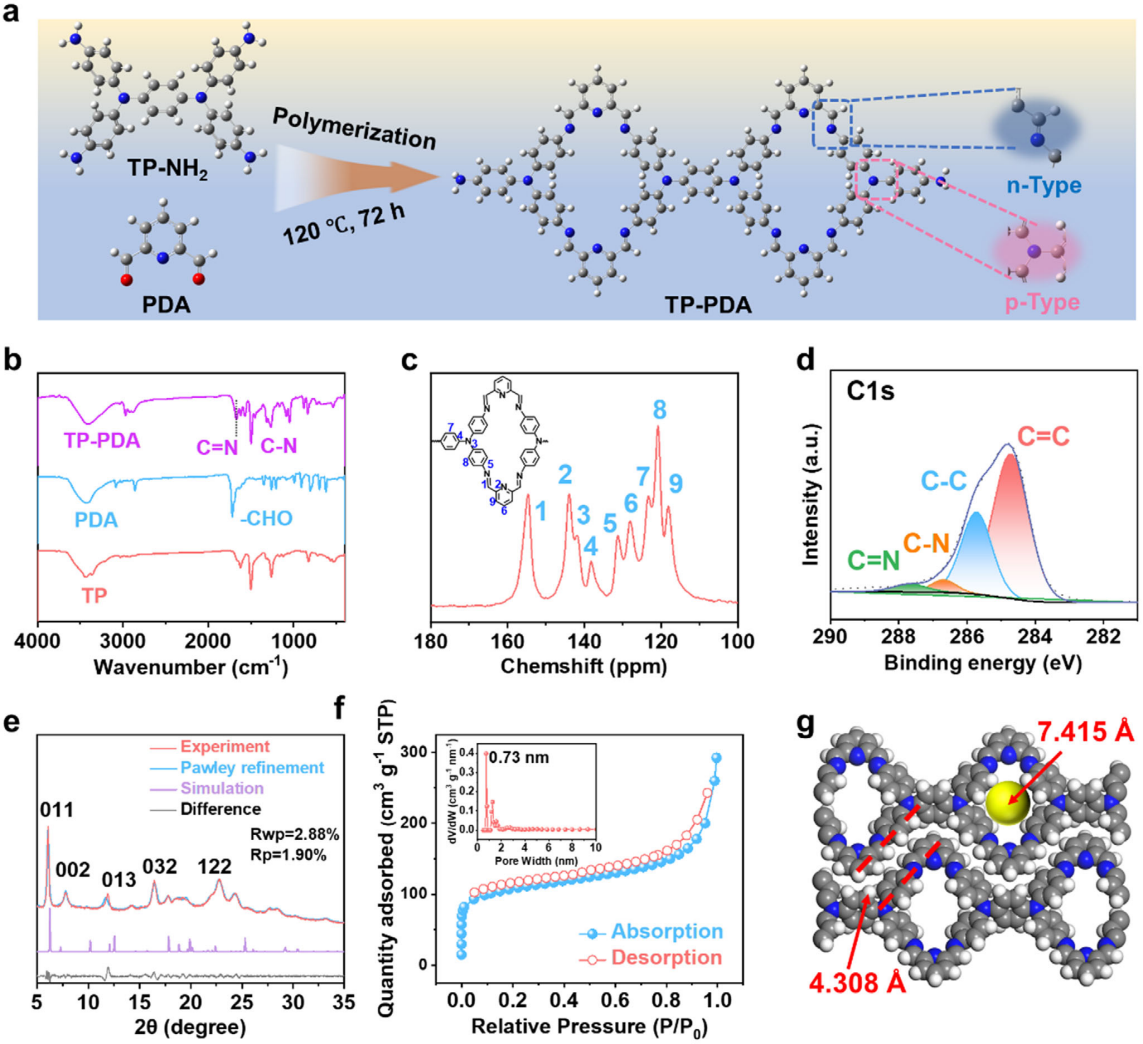
a) Schematic illustration of the synthetic process; b) FTIR spectrum; c) ^13^C NMR spectrum; d) C1s XPS spectrum; e) XRD patterns of the measured and simulated; f) N_2_ adsorption/desorption isotherm and pore size distribution; g) Simulation of aperture size.

In order to obtain electrode materials with stable properties, large theoretical capacity and high discharge voltage, *N*,*N*,*N*′,*N*′‐Tetrakis(4‐aminophenyl)‐1,4‐phenylenediamine (TP‐NH_2_), which contains two sp^3^ hybridization nitrogen (C─N) redox‐active centers and 2, 6‐pyridine dicarboxylic aldehyde (PDA) containing aldehyde groups were used as reactants. TP‐NH_2_ provides two sp^3^ N redox‐active centers, while PDA introduces aldehyde functional groups, enabling the formation of a 1D imine‐linked COF (TP‐PDA) through a Schiff base reaction (**Figure** **1**a). Detailed synthesis procedures are provided in the Supporting Information. The structural design of TP‐PDA enhances sodium‐ion storage by integrating both p‐type and n‐type redox‐active sites. Sp^3^ N as a p‐type redox center, facilitating anion binding and removal, while C═N bonds act as n‐type sites, enabling the insertion and extraction of Na^+^. This configuration establishes a dual‐ion storage mechanism, promoting enhanced electrochemical performance.

The successful synthesis of TP‐PDA was confirmed through multiple spectroscopic and structural characterization techniques. Fourier transform infrared spectroscopy (FTIR) (Figure 1b) reveals the disappearance of the ─CHO stretching vibration from PDA after polymerization, while a new C═N stretching peak at 1670 cm^−1^ confirms the formation of imine linkages. Further validation is provided by solid‐state ^13^C nuclear magnetic resonance (NMR) spectroscopy (Figure 1c), which exhibits a peak at 155 ppm, corresponding to the carbon atoms in C═N bonds. The spectrum presents nine distinct chemical shifts, consistent with the expected carbon environments in the TP‐PDA structure. The chemical composition and bonding environment were further examined using X‐ray photoelectron spectroscopy (XPS). The C1s spectrum (Figure 1d) displays distinct peaks for C═C, C─C, C─N, and C═N bonds, while the N1s spectrum (Figure S1b, Supporting Information) confirms the presence of C─N and C═N functionalities, reinforcing the formation of imine‐linked TP‐PDA.

Structural order in COF materials is a critical factor influencing ion transport and electrochemical performance. The crystallinity of TP‐PDA was examined using powder X‐ray diffraction (PXRD), which revealed sharp diffraction peaks at 6.08°, 7.80°, 11.88°, 16.44°, and 22.76° (Figure 1e). These reflections correspond to the (011), (002), (013), (032), and (122) lattice planes, respectively, indicating a highly ordered framework. Further refinement using structural simulations and Pawley analysis confirmed the P21/M symmetry, with optimized unit cell parameters of a = 4.52 Å, b = 17.22 Å, c = 24.01 Å, and *α* = *γ* = 90.0°, *β* = 94.24° (Rp = 1.90%, Rwp = 2.88%). The high degree of crystallinity suggests a well‐defined molecular stacking arrangement, which is expected to enhance charge transport and long‐term structural stability.

The morphological characteristics of TP‐PDA were examined using scanning electron microscopy (SEM) (Figure S2, Supporting Information). The COF exhibits a layered, sheet‐like structure, assembled into a flower‐like morphology. Elemental mapping reveals a uniform distribution of C and N, consistent with the expected molecular composition. The well‐defined sheet‐like stacking suggests effective molecular ordering, which is advantageous for maintaining structural integrity during repeated charge–discharge cycling. Porosity plays a crucial role in ion transport and charge storage, particularly in organic electrode materials. The nitrogen (N_2_) adsorption‒desorption isotherms (Figure 1f) indicate a microporous structure with a measured pore size of 0.73 nm, aligning closely with theoretical predictions from computational simulations (Figure 1g). The porous network facilitates efficient Na^+^ and anion diffusion, improving accessibility to redox‐active sites and enhancing overall charge transport. The well‐defined microporosity not only promotes rapid ion mobility but also contributes to improved electrolyte infiltration, which is essential for stable electrochemical cycling.

**Figure 2:**
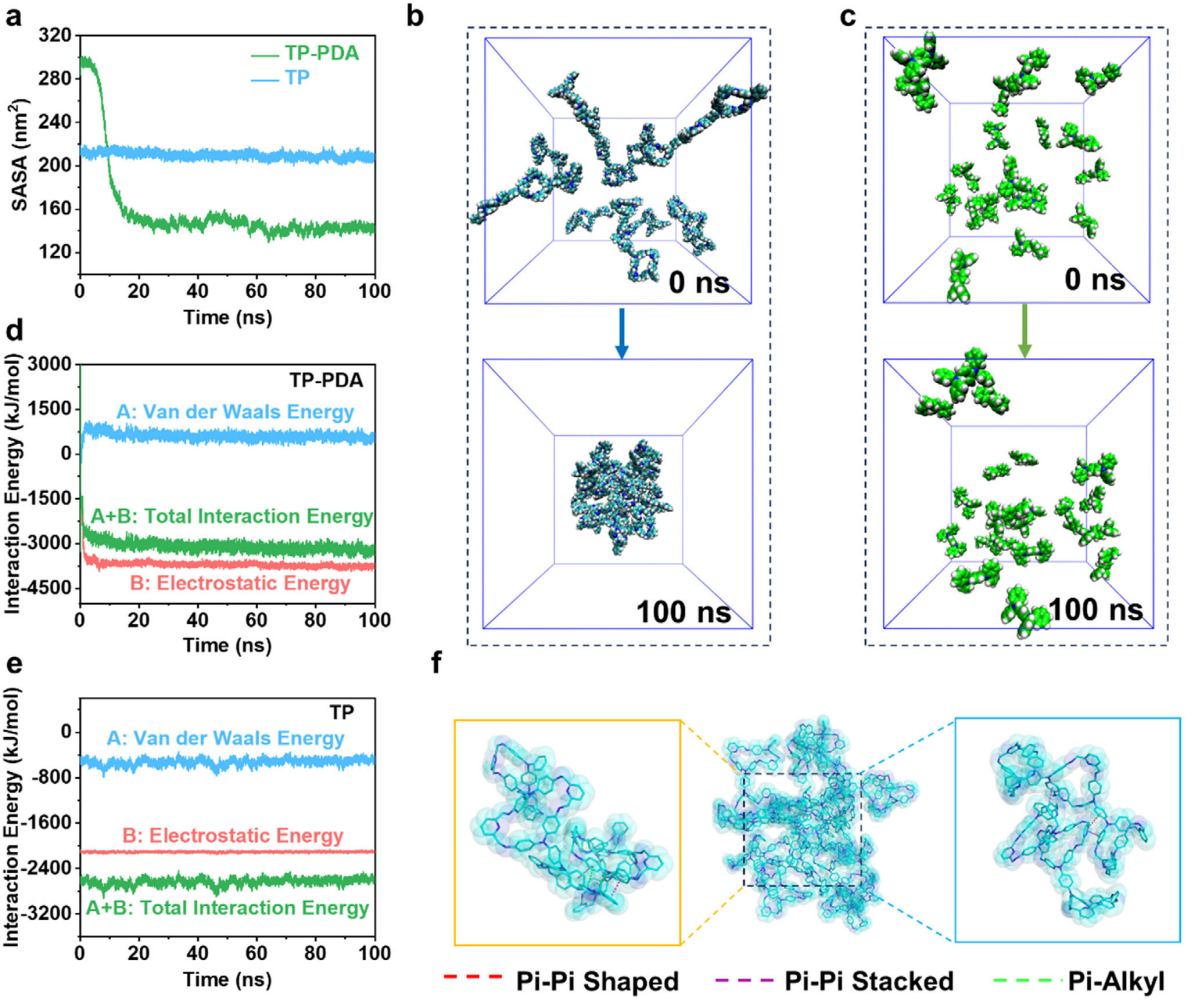
a) The variation of solvent accessible surface area (SASA) with simulation time in the COF and monomers systems. The variation of intermolecular binding energy with simulation time in (b) the COF and c) monomers systems. Structural changes between the initial and final states during the simulation process in d) the COF and e) monomers systems; f) Interaction patterns between molecules in the COF system.

The dissolution of organic electrode materials in organic electrolytes is a major challenge that significantly impacts the battery capacity and long‐term stability. Computational simulations were performed to investigate the structural stability and aggregation behavior of TP‐PDA COF materials in an electrolyte environment. The Solvent Accessible Surface Area (SASA) serves as a key parameter to evaluate the compactness of nanoparticles. As shown in **Figure** **2**a, the SASA values of the COF system exhibit a rapid decline in the early stages of the simulation, followed by a gradual decrease, eventually stabilizing after 20 ns. The average SASA value for the COF system is 159.467 ± 39.920 nm^2^, whereas the monomer system shows minimal fluctuation, maintaining an average SASA of 209.830 ± 2.816 nm^2^. The significant SASA reduction in the COF system suggests that TP‐PDA molecules self‐assemble into more compact nanostructures, thereby reducing the number of solvent‐exposed atoms.

The conformational changes and compactness of the self‐assembled structures before and after the simulation were further analyzed using cluster analysis in Gromacs. RMSD values were calculated over the 70 ‒ 100 ns simulation period, with a cutoff of 1.2 nm. The largest cluster in each system was extracted as the final conformations from the computational simulation (Figure 2b,c). The results indicate that TP‐PDA COFs self‐assemble into stable nanoclusters, whereas the monomer system remains dispersed. The dynamic aggregation process of small molecules was further visualized through representative conformations captured at 20 ns intervals (Figures S3 and S4, Supporting Information). Throughout the simulation, molecules within the COF system exhibit progressive aggregation, ultimately forming stable nanoparticle morphologies. These findings demonstrate that converting the TP monomer into a COF network effectively mitigates dissolution issues in organic electrolytes, thereby improving the structural stability of the electrode and enhancing its long‐term battery performance. UV–vis spectroscopy (Figure S5, Supporting Information) and Optical images of the TP‐PDA electrode in electrolyte (Figure S6, Supporting Information) further validate this behavior.

The driving forces governing molecular self‐assembly were analyzed by evaluating the interaction energy changes between molecules using the gmx_energy plugin (Figure 2d,e). Two primary interaction components were considered: Coul‐SR (electrostatic energy), which includes hydrogen bonding and ionic interactions, and LJ‐SR (van der Waals energy), primarily associated with *π*‒*π* stacking and other conjugation effects. In the COF system, the average Coul‐SR energy is ‒3778.241 kJ mol^−1^, while the average LJ‐SR energy is 604.705 kJ mol^−1^. Comparatively, in the monomer system, the Coul‐SR energy is significantly weaker (‒2108.703 kJ mol^−1^), and the LJ‐SR energy is ‒528.153 kJ mol^−1^. These results indicate that Coul‐SR interactions play a dominant role in stabilizing the COF self‐assembled structure. Further insights into molecular interaction patterns reveal that COF molecules engage in multiple non‐covalent interactions, including *π*‒*π* stacked conjugation, *π*‒*π* shaped interactions, and *π*‒alkyl interactions (Figure 2f). These intermolecular forces contribute to the stability and self‐assembly behavior of TP‐PDA COFs, ensuring their resistance to dissolution in electrolyte environments.

**Figure 3:**
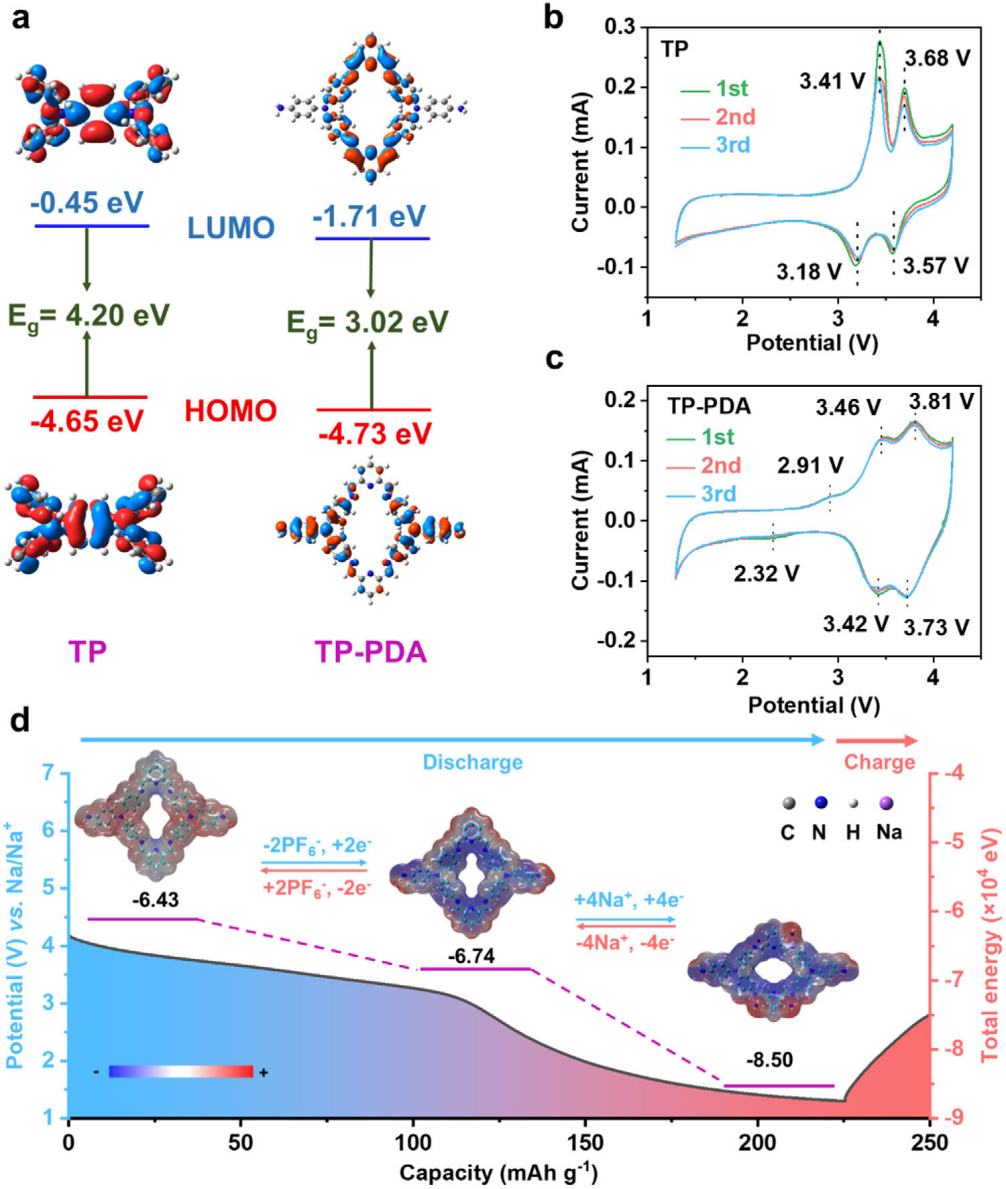
a) The LUMO‐HOMO plots of TP and TP‐PDA. The CV curves at 0.5 mV s^−1^ of b) TP cathode and c) TP‐PDA cathode. d) The molecular surface electrostatic potential and the total energy of TP‐PDA at different stages.

A deeper theoretical understanding of TP‐PDA can be obtained using density functional theory (DFT) calculations, which optimize the molecular structures of TP and TP‐PDA and determine their lowest unoccupied molecular orbital (LUMO) and highest occupied molecular orbital (HOMO) energy levels. The results showed that polymerization led to a lower HOMO energy level of ‒4.73 eV and a bandgap of 3.02 eV (**Figure** **3**a). It is well established that the voltage of p‐type electrodes is negatively correlated with the HOMO level, while the electronic conductivity is inversely proportional to the bandgap energy.^[^ ^48^, ^49^ ^]^ Based on these relationships, TP‐PDA exhibited enhanced electronic conductivity and a higher redox potential compared to the monomer.

The reduction peaks of TP‐PDA (3.73 and 3.42 V) were higher than those of TP (3.57 and 3.18 V) (Figure 3b,c). This shift was attributed to the extended conjugated system introduced through polymerization, which enhanced electron delocalization, increased redox potential and improved charge transport properties. The CV profile also revealed two pairs of peaks at 3.42/3.46 V and 3.73/3.81 V, which corresponded to the anion uptake and release at the sp^3^ N active centers. Additionally, TP‐PDA exhibited an extra pair of redox peaks at 2.32 and 2.91 V, attributed to the redox activity of C═N bonds, which is sodium‐ion insertion and extraction. These bipolar redox characteristics contributed to the high theoretical specific capacity of TP‐PDA (230 mAh g^−1^).

The molecular surface electrostatic potential was analyzed to further explore the charge distribution within TP‐PDA and its impact on ion binding and reaction pathways.^[^ ^50^, ^51^, ^52^, ^53^, ^54^ ^]^ This analysis is particularly important in electrochemical systems as it provides a fundamental understanding of ion adsorption sites, charge transfer mechanisms and molecular stability. As depicted in Figure 3d, in the initial state, the electron‐rich regions (blue zones) were concentrated around the C═N groups, indicating their strong affinity for Na^+^. Upon discharge, Na^+^ bound to the C═N sites, resulting in a localized increase in electrostatic potential in these regions. In the oxidized state, the sp^3^‐hybridized nitrogen atoms became electron‐deficient (red regions), suggesting a high electrophilic nature, which enhanced anion (PF_6_^−^) attraction. This confirmed that both cations (Na^+^) and anions (PF_6_^−^) actively participated in the charge–discharge process, further validating the dual‐ion storage mechanism of TP‐PDA. The relative stability of TP‐PDA during electrochemical cycling was further examined by calculating the total energies of its oxidized, initial and reduced states. The results showed a progressive decrease in total energy from ‒6.43 eV (oxidized) to ‒6.72 eV (initial) and ‒8.50 eV (reduced). This decreasing trend indicated that TP‐PDA became thermodynamically more stable as Na^+^ was inserted during discharge, reinforcing its suitability as a robust cathode material for sodium‐ion batteries.

### 2.2 Electrochemical Performance of Half Cell

**Figure 4:**
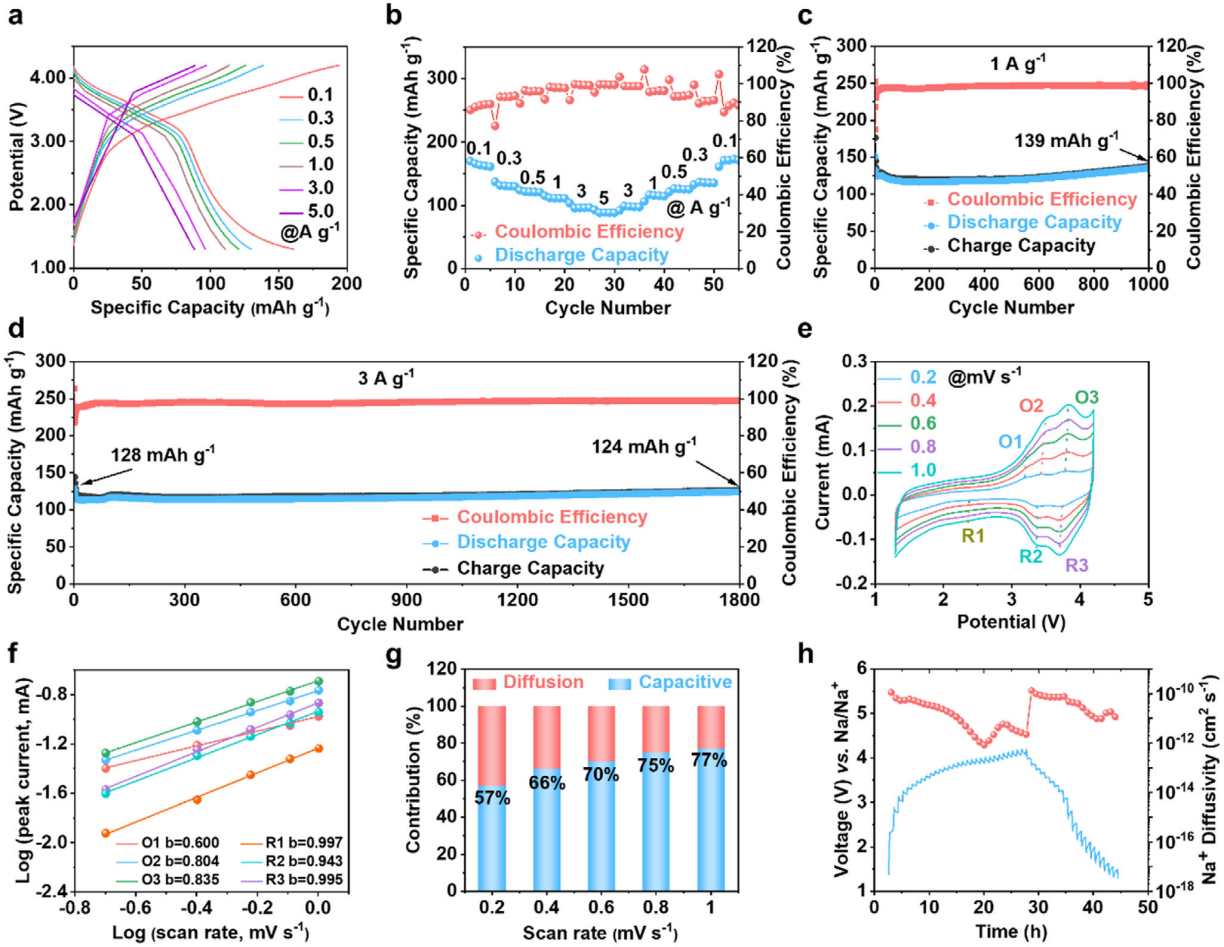
Electrochemical characterization of TP‐PDA in half‐cell configuration. a) Charge–discharge profiles at varying current densities; b) Rate capability assessment; c) Cycling performance at 1 A g^−1^; d) Cycling performance at 3 A g^−1^; e) CV curves at different scan rates; f) log(*i_p_*) versus log(*v*) plots at redox peaks for determining b‐values; g) Pseudocapacitive contribution analysis; h) GITT curves and ionic diffusion coefficient.

TP‐PDA was directly mixed with conducting carbon (Super‐P) and polyvinylidene fluoride (PVDF) to prepare electrodes without requiring any post‐processing. Its electrochemical performance as a cathode material for sodium‐ion batteries (SIBs) was evaluated in coin cells, with metallic sodium serving as the counter electrode (Detailed procedures are provided in the Supporting Information). The rate capability of TP‐PDA was assessed by measuring the discharge capacity at various current densities (**Figure** **4**a). At 0.1 A g^−1^, TP‐PDA delivered a high discharge capacity of 175 mAh g^−1^ and an average discharge potential of 3.1 V, yielding an energy density of 542 Wh kg^−1^. Despite a gradual decline with increasing current density, it retained 90 mAh g^−1^ at 5 A g^−1^, demonstrating exceptional rate performance. Upon reducing the current density back to 0.1 A g^−1^, the discharge capacity recovered to its initial state (Figure 4b), indicating high structural stability and rapid charge transfer properties. This rate capability is attributed to the porous layered structure, which facilitates ion transport and charge storage. It is noteworthy that the actual capacity of TP‐PDA still shows a certain gap compared to its theoretical capacity of 230 mAh g^−1^. To address this issue, the integration of COF materials with carbon‐based materials (such as carbon nanotubes) can be employed to develop organic‐inorganic hybrid composites. This approach can further enhance the charge transfer efficiency within the electrode, potentially enabling the achievement of the theoretical capacity.^[^ ^55^ ^]^

Long‐term cycling stability was further evaluated at 1 and 3 A g^−1^. TP‐PDA retained a high discharge capacity of 139 mAh g^−1^ after 1000 cycles at 1 A g^−1^ and 124 mAh g^−1^ after 1800 cycles at 3 A g^−1^, with a capacity retention exceeding 90% in both cases (Figure 4c,d). At the same time, Electrochemical Impedance Spectroscopy (EIS) showed little change in the impedance of the battery in the initial state and after the cycle, which indicates that the battery has good stability (Figure S8, Supporting Information). The charge–discharge curves exhibited well‐defined voltage plateaus (Figures S9 and S10, Supporting Information), highlighting the excellent electrochemical reversibility and cycling durability of TP‐PDA. These performance metrics outperform those of many previously reported COF‐based electrodes (Table S5, Supporting Information), demonstrating its superior cycling stability and fast redox kinetics.

For further investigating the charge storage mechanism and electrochemical kinetics, cyclic voltammetry (CV) was performed at varying scan rates (Figure 4e). The minimal potential shift between the cathodic and anodic peaks indicates that the TP‐PDA electrode exhibits favorable redox kinetics. Additionally, the peak current increased nearly linearly with the scanning rate. Using the equation:^[^ ^56^ ^]^

(1) $$i_{p}=av^{b}\;$$

where *i_p_* is the peak current and *ν* is the scanning rate, all calculated b values are >0.5 and approach 1.0 (Figure 4f). This suggests that the TP‐PDA electrodes, characterized by ultrafast redox kinetics, operate through a charge storage mechanism predominantly governed by capacitive. The capacitive contribution of the TP‐PDA electrode is quantified in Figures S11–S15 (Supporting Information) using the formula:^[^ ^57^ ^]^

(2) $$i=k_{1}v+k_{2}v^{1/2}$$

where *k_1_ν* and *k_2_ν* ^1/2^ represent the contributions from surface reactions and diffusion processes, respectively. As the scan rate increased, the capacitive contribution increased from 57% at 0.2 mV s^−1^ to 77% at 1 mV s^−1^ (Figure 4g), confirming that pseudocapacitive charge storage dominates under high‐rate conditions, enabling rapid charge–discharge cycling. The ionic diffusion coefficient was determined using the Galvanostatic Intermittent Titration Technique (GITT).^[^ ^58^, ^59^ ^]^ A pulse current of 100 mA g^−1^ was applied for 10 min, followed by a 40 min relaxation period to reach equilibrium. The resulting diffusion coefficient of TP‐PDA ranged from 10^−10^ to 10^−12^ cm^2^ s^−1^ (Figure 4h), aligning with typical values observed for COF‐based electrodes in sodium‐ion batteries. This high ionic diffusion coefficient reinforces TP‐PDA's capability to facilitate rapid ion transport, making it a promising candidate for high‐power SIB applications.

### 2.3 Redox Mechanism

**Figure 5:**
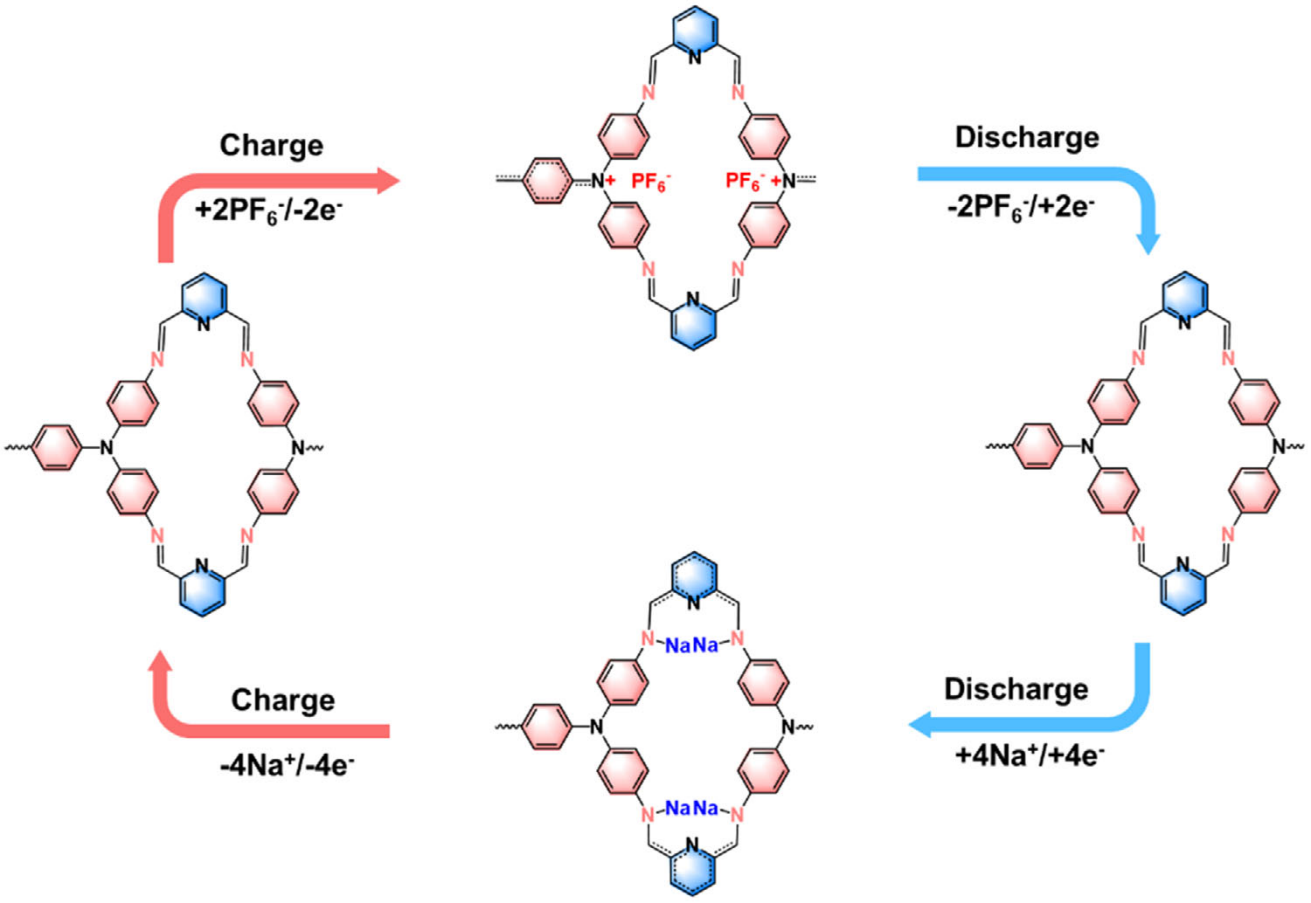
Redox reaction mechanism of TP‐PDA.

The TP‐PDA structure incorporates both n‐type (C═N) and p‐type (sp^3^ N) redox‐active groups, classifying it as a bipolar material. The C═N groups facilitate the reversible insertion and extraction of Na^+^, while the sp^3^ N groups enable the reversible binding and release of PF_6_^−^. The charge–discharge process follows a four‐stage mechanism: During charging, TP‐PDA undergoes oxidation, losing electrons and incorporating PF_6_^−^ to maintain charge neutrality. In the subsequent step, a reduction reaction occurs, releasing PF_6_^−^ and restoring TP‐PDA to its original state. Subsequently, the C═N groups undergo reduction, leading to the incorporation of four Na^+^ ions. Finally, during recharging, oxidation removes the Na^+^ ions, returning TP‐PDA to its initial form (**Figure** **5**). This process comprises two oxidation and two reduction steps, enabling each structural unit to cycle six electrons, resulting in a theoretical specific capacity of 230 mAh g^−1^.

**Figure 6:**
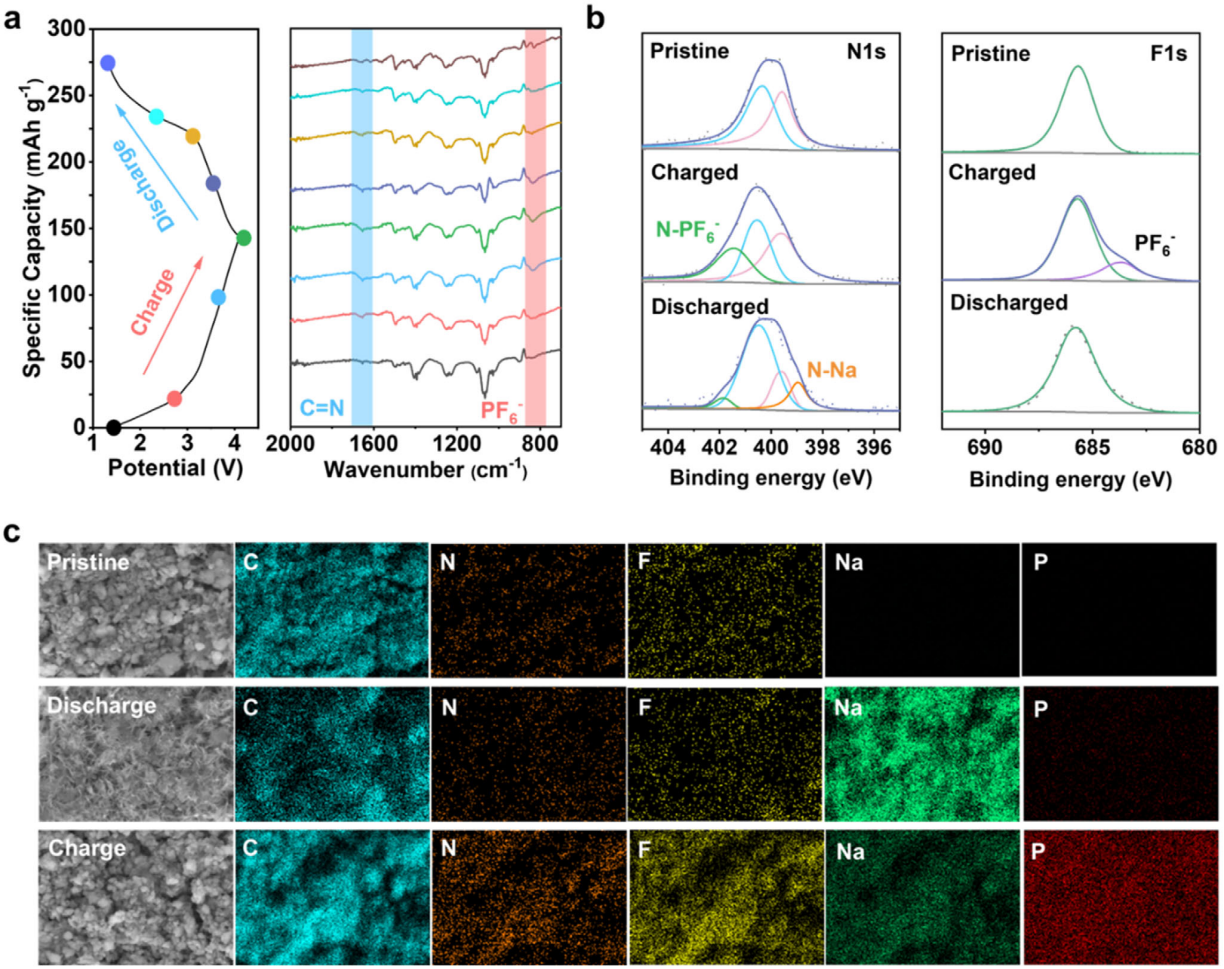
a) FTIR spectrum, b) XPS spectrum, and c) SEM image and mapping at different charge and discharge stages.

The reversible redox process was confirmed by Fourier transform infrared spectroscopy (FTIR) (**Figure** **6**a). At the lowest discharge voltage (1.3 V), the absorption peak associated with the C═N stretching vibration at 1655 cm^−1^ completely disappeared. Upon charging, the C═N signal gradually reappeared and intensified, whereas during the discharging process, the C═N signal progressively weakened and eventually disappeared again. This variation of the infrared absorption peak corresponding to the C═N bond clearly demonstrates the reversible insertion and extraction of Na^+^ ions at the C═N sites, functioning as n‐type redox centers. Simultaneously, the absorption peak corresponding to the N–PF_6_^−^ group exhibited a similar reversible behavior, confirming that the sp^3^‐hybridized nitrogen atoms, serving as p‐type active sites, reversibly accommodate PF_6_^−^ anions. Collectively, these FTIR observations strongly support a fully reversible dual‐ion (Na^+^ and PF_6_^−^) insertion‐extraction mechanism in TP‐PDA during cycling.

The ion transport and Na^+^/PF_6_^−^ insertion–extraction processes were examined using ex situ X‐ray Photoelectron Spectroscopy (XPS) on TP‐PDA electrodes at different charge–discharge states: initial, fully charged, and fully discharged. The C1s XPS spectrum shows that the C═N bond signal weakens during discharge, while the C–N peak intensity increases (Figure S16, Supporting Information). Upon charging, the peak intensities return to their initial states, confirming the reversible interaction between C═N and Na^+^. Similarly, the N1s XPS spectrum displays two primary peaks in the initial state, corresponding to C─N and C═N bonds (Figure 6b). A new peak appears upon charging, attributed to N–PF_6_^−^, confirming the incorporation of PF_6_^−^. After discharge, the N–PF_6_^−^ peak diminishes, indicating PF_6_^−^ removal, while a new N─Na peak emerges, signifying Na^+^ binding to C═N as an n‐type redox site. The F1s spectrum initially exhibits a single peak. After charging, a new peak appears, corresponding to PF_6_^−^ incorporation, which subsequently disappears upon discharge, reinforcing the reversible PF_6_^−^ intercalation/de‐intercalation process. The P2p XPS spectrum follows a similar trend, supporting the dual‐ion mechanism (Figure S17, Supporting Information). Additional XPS data, including XPS survey spectra and Na1s spectra at different charge–discharge stages, are provided in Figures S18–S21 (Supporting Information).

The morphological evolution and elemental distribution of TP‐PDA electrodes were analyzed using scanning electron microscopy (SEM) and energy‐dispersive X‐ray spectroscopy (EDS) (Figure 6c). The initial electrode surface exhibits a rough and porous morphology, with C, N, and F elements uniformly distributed, where F originates from the PVDF binder. Upon discharge, Na^+^ incorporation leads to pore coverage, as evidenced by a strong Na signal in EDS analysis. After full charging, the electrode morphology returns to its initial state, while the F and P signals increase and the Na content decreases, confirming Na^+^ extraction and its pairing with PF_6_^−^ during charging. These findings verify the dual‐ion charge–discharge mechanism in TP‐PDA. The quantitative elemental compositions at different states are summarized in Tables S2–S4 (Supporting Information).

### 2.4 Electrochemical Performance of the Full Battery

**Figure 7:**
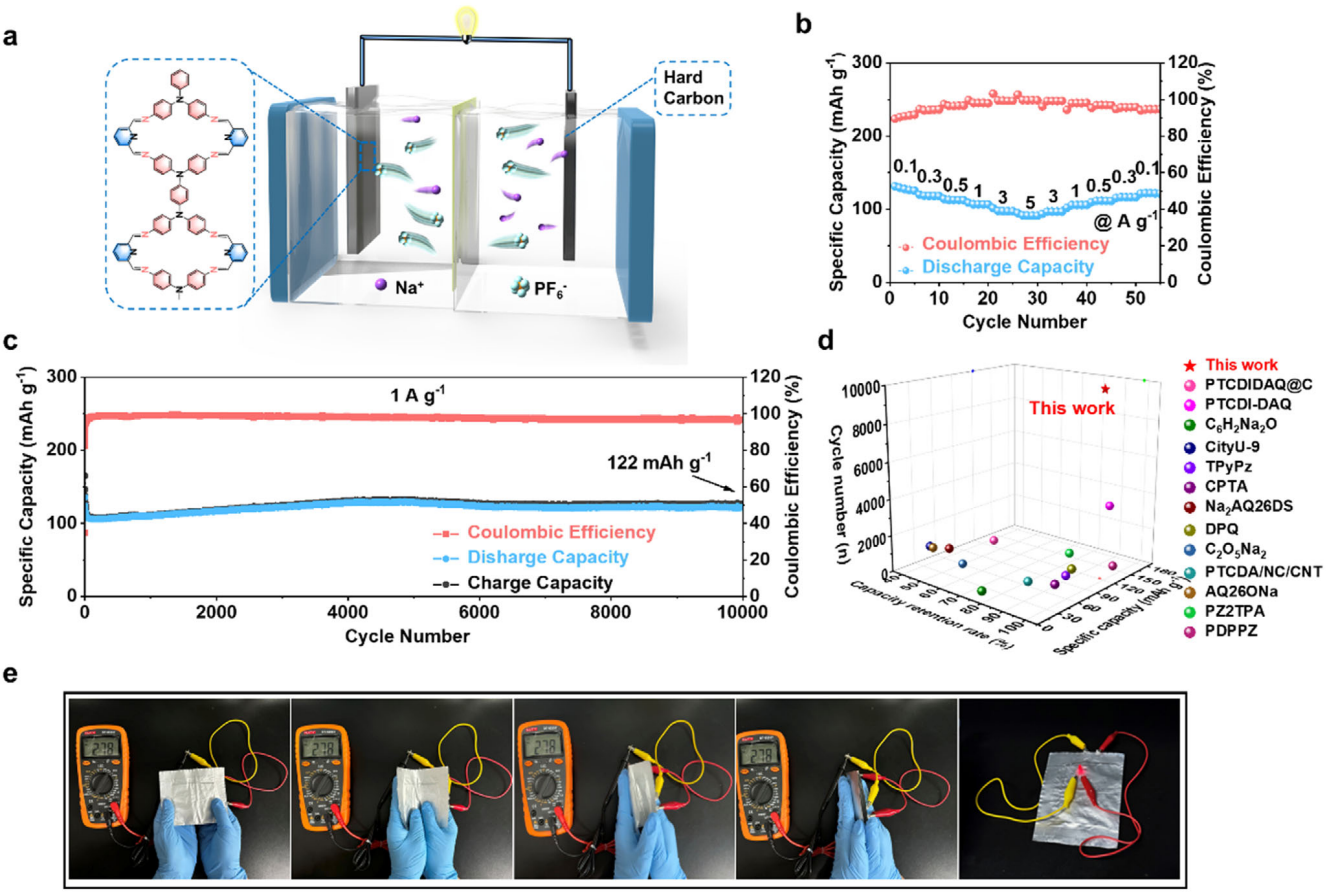
Electrochemical performance and mechanical flexibility of the TP‐PDA||HC full battery. a) Schematic representation of the full‐cell configuration; b) Rate capability at various current densities; c) Long‐term cycling stability at a current density of 1 A g^−1^; d) Comparison of electrochemical properties of organic full battery; e) Galvanostatic charge–discharge profiles; f) Photographs of the flexible pouch cell under different bending angles, demonstrating stable voltage retention and continuous LED operation.

The practical feasibility of TP‐PDA as a cathode material was evaluated in a full sodium‐ion battery, assembled with TP‐PDA as the cathode and hard carbon (HC) as the anode. A schematic illustration of the battery configuration is shown in **Figure** **7**a. During discharge, PF_6_^−^ ions are released from the cathode into the electrolyte, while Na^+^ ions intercalate into TP‐PDA, reducing it to a lower oxidation state. Simultaneously, at the anode, Na^+^ ions are extracted from HC and migrate through the electrolyte toward the cathode, while PF_6_^−^ moves toward the anode. The charge process follows the reverse mechanism, with Na^+^ ions deintercalating from TP‐PDA and PF_6_^−^ re‐entering the cathode.

The charge–discharge process involves the simultaneous transport of both cations and anions, classifying the system as a dual‐ion battery. The cyclic voltammetry (CV) profile of the TP‐PDA||HC full cell closely resembles that of the half‐cell, indicating that the combination of TP‐PDA and hard carbon maintains electrochemical compatibility. However, a shift in the redox peaks toward lower potentials is observed in the full‐cell CV curve (Figure S22, Supporting Information). This shift is attributed to the characteristics of hard carbon, which, as a negative electrode material in sodium‐ion batteries, operates over two distinct voltage regions: a sloping region from 1.1 to 0.1 V and a plateau region below 0.1 V. The CV profile of hard carbon is provided in Figure S23 (Supporting Information).

The rate capability of TP‐PDA||HC was also investigated (Figure 7b). At a current density of 0.1 A g^−1^, the battery delivers a capacity of 125 mAh g^−1^. While the capacity declines slightly with increasing current density, it remains 80 mAh g^−1^ at 5 A g^−1^, demonstrating robust charge storage kinetics. This excellent rate performance is attributed to the porous structure of TP‐PDA, which facilitates efficient ion transport and redox activity. These findings highlight the TP‐PDA's potential as a high‐performance organic cathode material for next‐generation sodium‐ion batteries.

The cycling performance of TP‐PDA||HC was further assessed at varying current densities. After 1000 cycles at 0.5 A g^−1^, the full‐battery retains a discharge capacity of 140 mAh g^−1^ (Figure S24, Supporting Information). The charge–discharge profiles remain highly symmetrical, exhibiting well‐defined plateaus (Figure S25, Supporting Information). Increasing the current density to 1 A g^−1^ results in a slight capacity reduction in the initial cycles, possibly due to minor instability following cell assembly. However, after stabilization, the battery maintains a capacity of 122 mAh g^−1^, with no observable degradation after 10 000 cycles (Figure 7c), highlighting its exceptional long‐term stability and reversibility (Figure S26, Supporting Information). And, as far as we know, this organic sodium ion full battery is one of the best performances of all full batteries reported (Figure 7d).

Beyond electrochemical performance, the mechanical flexibility of the TP‐PDA||HC pouch cell was evaluated by measuring voltage retention under different bending conditions (Figure 7e). When bent at 0°, 90°, 120°, and 180°, the pouch battery maintains a stable voltage of 2.78 V, with no noticeable voltage drop. Additionally, the LED powered by the flexible battery continues to glow consistently, confirming its mechanical resilience and suitability for flexible and wearable electronic devices.

## 3 Conclusion

A 1D covalent organic framework (COF), TP‐PDA, was successfully synthesized via a Schiff base reaction between TP‐NH_2_ and PDA. The material integrates dual‐redox (n‐type and p‐type) active centers, enabling sodium‐ion and anion co‐storage, which enhances charge transfer kinetics and electrochemical stability. Both theoretical calculations and experimental results confirm that TP‐PDA exhibits a higher reduction potential and improved conductivity compared to its monomer precursor, while also demonstrating insolubility in organic electrolytes, ensuring long‐term cycling stability.

The linear and porous structure facilitates efficient ion transport, contributing to high electrochemical performance without requiring post‐synthesis modifications. TP‐PDA delivers an average discharge potential of 3.1 V. In sodium half‐cells, the material maintains 139 mAh g^−1^ after 1000 cycles at 1 A g^−1^ and 124 mAh g^−1^ after 1800 cycles at 3 A g^−1^, with a capacity retention exceeding 96%. When paired with a hard carbon anode in a full‐cell configuration, the TP‐PDA||HC battery achieves stable cycling over 10 000 cycles at 1 A g^−1^ with negligible capacity degradation.

Beyond electrochemical performance, TP‐PDA also demonstrates mechanical resilience, as evidenced by the pouch cell maintaining stable voltage under bending conditions. These results establish TP‐PDA as a promising candidate for high‐performance organic cathodes in next‐generation sodium‐ion batteries, particularly for flexible and wearable energy storage applications.

## Conflict of Interest

The authors declare no conflict of interest.

## Supporting information



Supporting Information

## Data Availability

The data that support the findings of this study are available from the corresponding author upon reasonable request.
