# Supplementary material for: High Voltage Flexible Sodium‐Ion Battery Cathode Materials Based on 1D Covalent Organic Framework
Source: Adv Sci (Weinh). 2025 Jun 23;12(31):e05311. doi: 10.1002/advs.202505311 (PMC12376503; doi:10.1002/advs.202505311)
Supplement: Supplementary file 1 — Supporting Information [file ADVS-12-e05311-s001.docx]

Supporting Information

**High Voltage Flexible Sodium-Ion Battery Cathode Materials Based on One-Dimensional Covalent Organic Framework**

*Shuai Liu, Puiki Leung*, Yong Zuo, Meng Sun, Lei Wei*, Frank C Walsh, Tianshou Zhao*, and Qiang Liao*

**Section S1. Materials and Methods**

**Synthesis of TP-PDA**

All reagents and solvents are purchased from commercial suppliers and used directly without further purification.

The TP-PDA was synthesized using a solvothermal method. *N,N,N',N'*-Tetrakis(4-aminophenyl)-1,4-phenylenediamine (TP-NH_2_, 0.05 mmol, 23.63 mg) and 2,6-Pyridinedicarboxaldehyde (PDA, 0.10 mmol, 13.51 mg) were suspended in a mixture of o-dichlorobenzene and n-butyl alcohol (1:1 v/v, 4 mL). The resulting mixture was subjected to sonication for 30 minutes, after which aqueous acetic acid (3 M, 0.4 mL) was added. Following this, the mixture underwent three freeze-pump-thaw cycles, and the ampoule tubes were sealed under vacuum and heated at 120 °C for 3 days. The TP-PDA precipitate was then isolated via vacuum filtration and washed sequentially with DMAc, DMF, and tetrahydrofuran. Further purification was achieved through Soxhlet extraction with tetrahydrofuran, methanol, and acetone for 48 hours. Finally, the product was dried at 60 °C under vacuum for 24 hours, yielding a brownish-yellow powder.

**Materials characterizations**

These are the characterization instruments used in this experiment. Fourier transform infrared (FTIR, Thermo Fisher Scientific Nicolet iS20, America), Nuclear magnetic resonance (NMR, Bruker Avance Neo 400WB, Germany), X-ray photoelectron spectrometer (XPS, Thermo Scientific K-Alpha, America). X-ray diffractometer (XRD, Rigaku SmartLab SE, Japan), Accelerated Surface Area and Porosimetry System (BET, Micromeritics ASAP 2460, America), Scanning electron microscopy (SEM, TESCAN MIRA LMS, Czech Republic).

**Structural modelling**

Structural atomistic simulations of the possible framework structures were carried out using Material Studio software. The simulated PXRD patterns were determined by the Reflex module.

**Molecular dynamics simulation**

A simulation box with dimensions of 6 nm × 6 nm × 6 nm was initially constructed, randomly filled with 10 COF molecules and 20 MOL molecules to form the simulation systems. Molecular dynamics (MD) simulations were performed using Gromacs 2022.2 under constant temperature and pressure with periodic boundary conditions.^[1]^ The GAFF all-atom force field was applied, and all systems were solvated with propylene carbonate.^[2]^ During the MD simulations, hydrogen bonds were constrained using the LINCS algorithm,^[3]^ with an integration time step of 2 fs. Electrostatic interactions were computed using the Particle-Mesh Ewald (PME) method.^[4]^ Non-bonded interaction cutoffs were set to 10 Å, updated every 10 steps. Temperature control at 298.15 K was achieved via the V-rescale coupling method,^[5]^ while pressure was maintained at 1 bar using the Parrinello-Rahman method.^[6]^ Initially, energy minimization was carried out for all four systems using the steepest descent algorithm to eliminate close atomic contacts. Subsequently, a 100 ps NVT equilibration simulation was conducted at 298.15 K. Finally, 100 ns MD simulations were performed for two distinct systems, with conformations saved every 10 ps. Visualization of simulation results was completed using the built-in Gromacs tools and VMD.

**Electrode preparation**

The electrode is prepared by mixing TP-PDA, super p, PVDF binder according to the ratio of 5:4:1 and adding NMP to prepare a uniform slurry. Apply the paste evenly to the aluminum foil collector with carbon layer. Vacuum 80 degrees after 12 hours cut into a diameter of 12 mm circular electrode. The load of one electrode sheet is 1—2 mg. At the time of ex situ testing, the mass ratio of TP-PDA, Super p, PVDF on the electrode was 7:2:1.

**Battery assembly**

The battery model used in the lab is CR2032. In the half battery, the negative electrode uses sodium metal, the electrolyte is 1 M NaPF_6_-PC, the diaphragm is glass fiber membrane GF/D, and the positive electrode uses TP-PDA. After the electrode assembly was completed, the electrochemical performance was tested after standing for 8 hours. All tests were carried out in a 25℃ incubator. When assembling a full battery, the anode material is hard carbon, and the other materials are the same as the half battery. When assembling the pouch battery, the cathode and anode are cut to the designed dimensions (40×60 mm) with tabs retained, and stacked in the sequence of anode-separator-cathode. The aluminum laminate film (three-layer structure: PET/Al/PP) is cut and folded into a pouch shape, with three edges heat-sealed. The 1M NaPF_6_-PC electrolyte is injected, followed by 1-2 hours standing for complete infiltration. The electrolyte-filled cell is then placed in a vacuum sealer to remove residual gas bubbles before finally heat-sealing the fourth edge to ensure leak-free encapsulation.

**Electrochemical characterizations**

The cyclic voltammetry (CV) and electrochemical impedance spectroscopy (EIS) were performed on the DH7003 electrochemical workstation. Rate performances, galvanostatic charge-discharge (GCD), galvanostatic intermittent titration technique (GITT) tests, were conducted on the Neware MHW-100-2-160CH test system.

**Density functional theory**

Density functional theory (DFT) calculations were performed using Gaussian 09 software. The geometric configuration and frequency of organic molecules at different discharge states were calculated at the B3LYP/6-31G (d, p) level. The molecule energy is optimized by the B3LYP/6-311G (d, p) basis set.

**The calculation of theoretical capacity**

Theoretical capacity C (mAh g^-1^) was calculated using the following equation:

$$C=\frac{nF}{3600(Mw/1000)}$$

Where n and Mw is the number of charge carrier and the molecular weight of the active material, respectively, F is the Faraday constant (96485 C/mol). The molecular weight of a repeating unit is calculated to be Mw = 700.85 g/mol. The number of electrons (n) involved in the repeating unit is 6. Therefore, the theoretical capacity is calculated using the equation of C=229 mAh g^-1^.


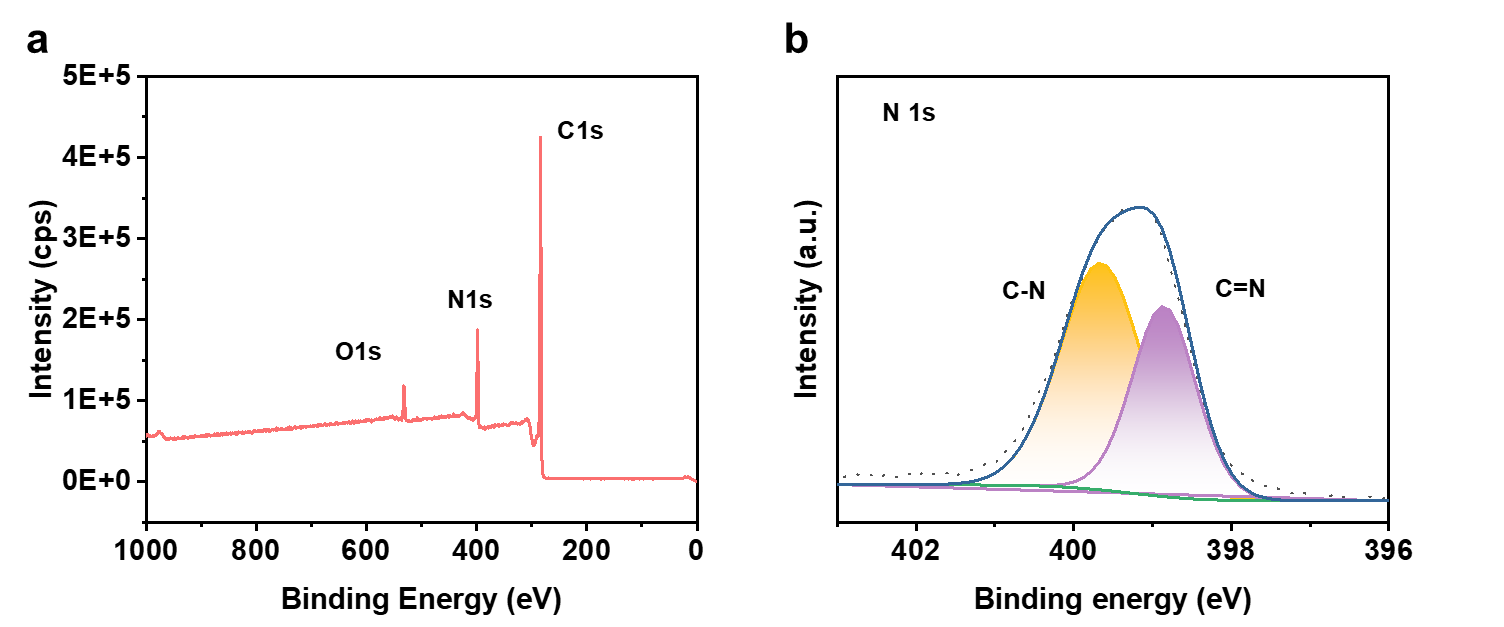


Figure S1. (a) XPS survey spectrum. (b) N1s of XPS spectrum.


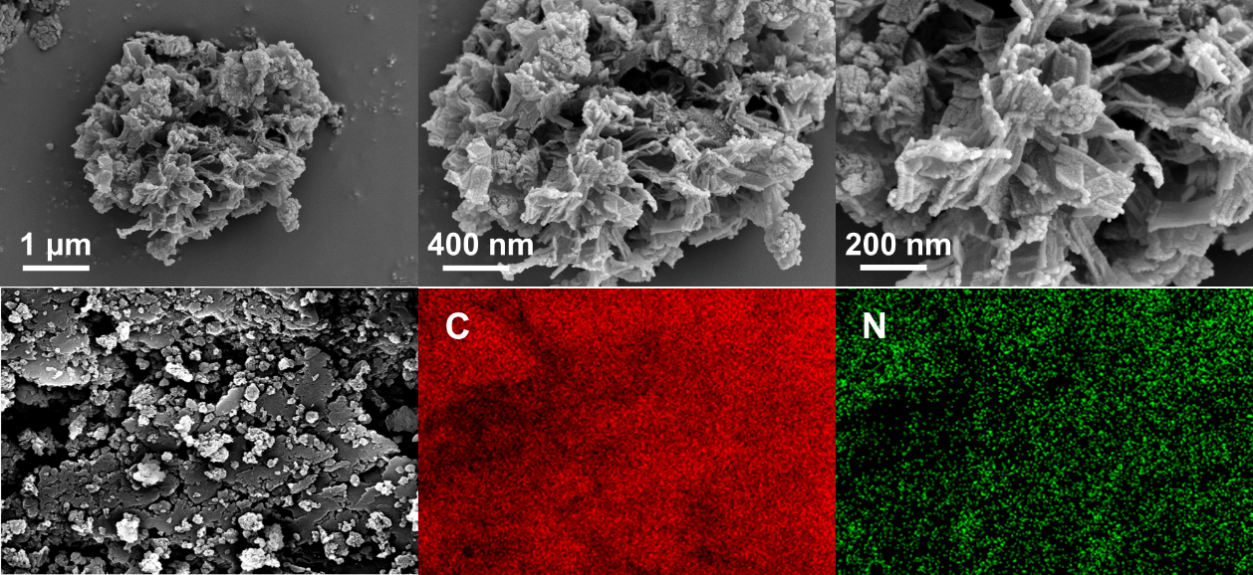


Figure S2. SEM image and element distribution of TP-PDA.


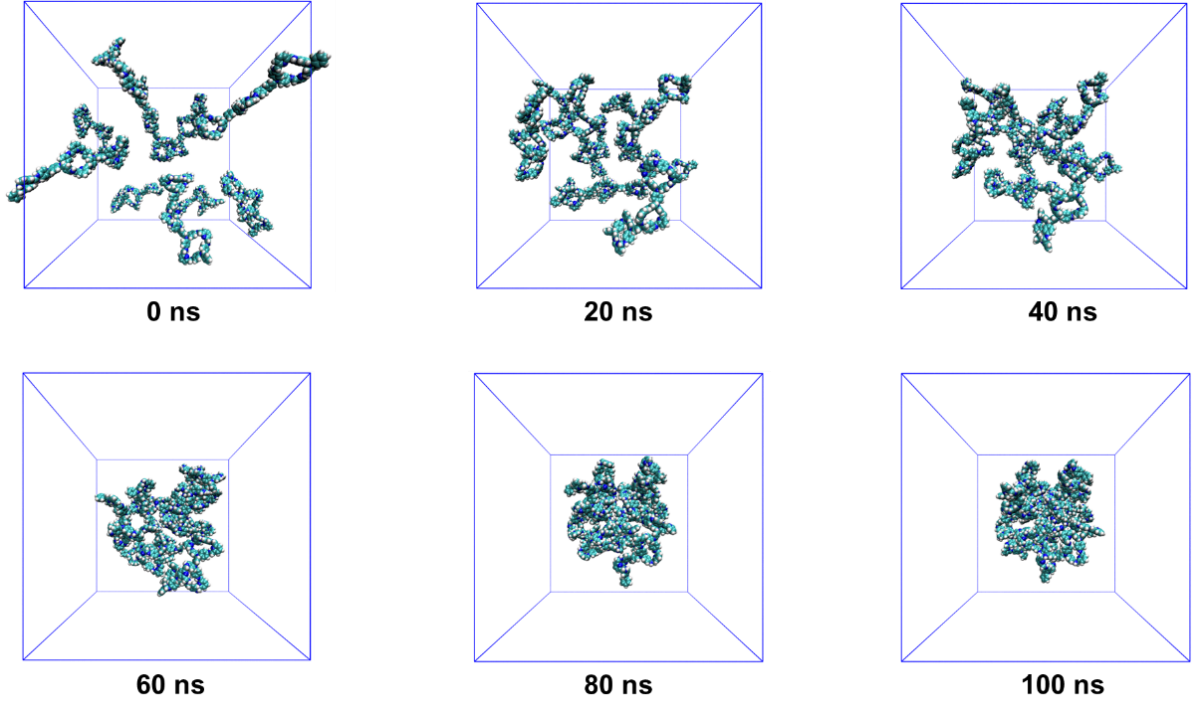


Figure S3 Temporal structural changes in the TP-PDA COF system throughout the simulation.


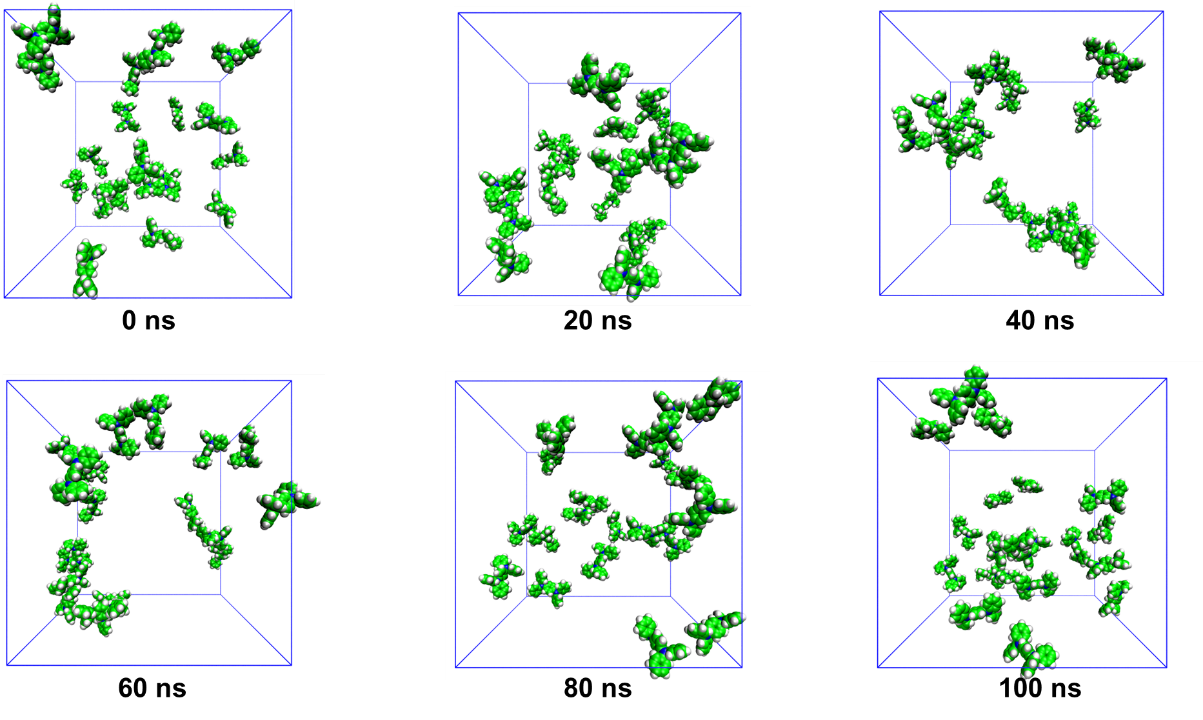


Figure S4 Temporal structural changes in the TP monomers system throughout the simulation.

Figure S5 UV-vis spectroscopy of TP-PDA in PC, TP in PC and PC solvent.


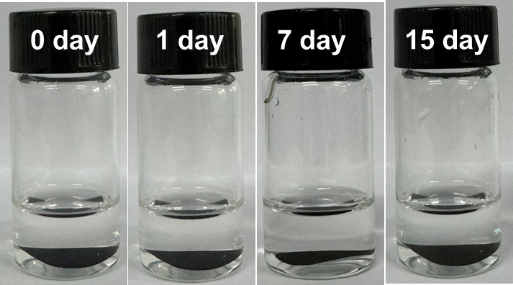


Figure S6. Photo of TP-PDA electrode immersed in electrolyte.

Figure S7. Cyclic performance of TP-PDA at a current density of 0.1 A g^-1^.

Figure S8. Nyquist plots for TP-PDA electrodes before and after cycling at the current density of 1.0 A g^-1^.

Figure S9. Charge and discharge curve at a current density of 1 A g^-1^.

Figure S10. Charge and discharge curve at a current density of 3 A g^-1^.

Figure S11. Contribution of the capacitance control at 0.2 mV s^−1^.

Figure S12. Contribution of the capacitance control at 0.4 mV s^−1^.

Figure S13. Contribution of the capacitance control at 0.6 mV s^−1^.

Figure S14. Contribution of the capacitance control at 0.8 mV s^−1^.

Figure S15. Contribution of the capacitance control at 1.0 mV s^−1^.

Figure S16. C1s XPS spectrum of TP-PDA electrode in different charging and discharging stages.

Figure S17. P2p XPS spectrum of TP-PDA electrode in different charging and discharging stages.

Figure S18. XPS survey spectrum of TP-PDA electrode in the initial state.

Figure S19. XPS survey spectrum of TP-PDA electrode in a fully charged state.

Figure S20. XPS survey spectrum of TP-PDA electrode in a fully discharged state.


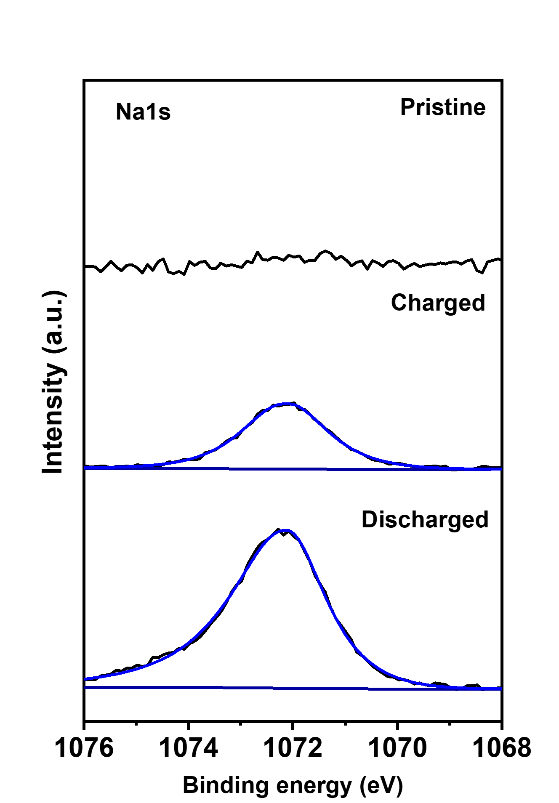


Figure S21. Na 1s XPS spectrum of TP-PDA electrode in different charging and discharging stages.

Figure S22. CV curves of TP-PDA||HC full battery


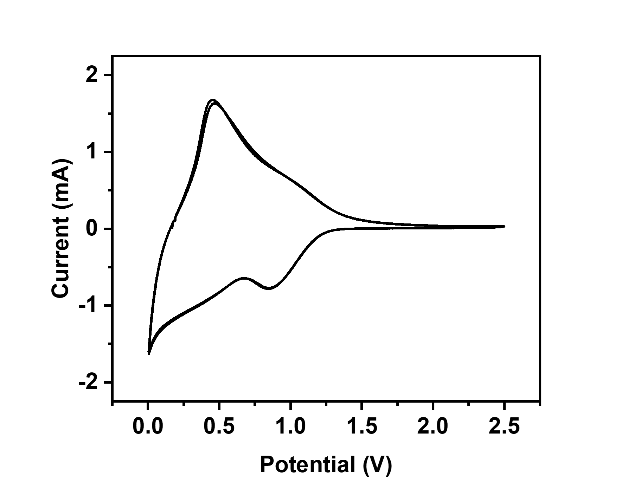


Figure S23. CV curves of Hard Carbon

Figure S24. TP-PDA||HC full battery cycle performance at a current density of 0.5 A g^-1^

Figure S25. Charge and discharge curves of TP-PDA||HC full battery at a current density of 0.5 A g^-1^.

Figure S26. Charge and discharge curves of TP-PDA||HC full battery at a current density of 1 A g^-1^.

Table S1. Fractional atomic coordinates for the unit cell of TP-PDA

| Space group P21/M | | | | | | | |
| --- | --- | --- | --- | --- | --- | --- | --- |
| a=4.527583 Å b=17.224119 Å c=24.005604 Å | | | | | | | |
| α=90.000° β=94.24292° γ=90.000° | | | | | | | |
| Atom | X | Y | Z | Atom | X | Y | Z |
| H | 0.007111 | 1.125319 | 0.554506 | C | -0.29685 | 1.680666 | -0.49884 |
| H | 0.284856 | 1.054506 | 0.48136 | C | -0.38823 | 1.608996 | -0.47172 |
| H | 0.350168 | 0.965326 | 0.417559 | C | -0.65135 | 1.555857 | -0.3941 |
| H | 0.429412 | 0.882223 | 0.337973 | C | -0.50818 | 1.48347 | -0.3887 |
| H | 1.050775 | 1.047587 | 0.280053 | C | -0.55195 | 1.435777 | -0.34283 |
| H | 0.966827 | 1.131975 | 0.359185 | C | -0.73922 | 1.459289 | -0.30182 |
| H | 0.987366 | 0.820775 | 0.330906 | C | -0.89931 | 1.528743 | -0.31009 |
| H | 1.492864 | 1.12532 | -0.05452 | C | -0.85381 | 1.576583 | -0.35549 |
| H | 1.21517 | 1.054507 | 0.018632 | C | -0.88393 | 1.290516 | -0.29533 |
| H | 1.149965 | 0.965334 | 0.082444 | C | -1.40821 | 1.679613 | 0.039645 |
| H | 1.070788 | 0.882232 | 0.162034 | C | -1.20316 | 1.680666 | -0.00116 |
| H | 0.44938 | 1.04758 | 0.219958 | C | -1.11181 | 1.608996 | -0.02828 |
| H | 0.533267 | 1.131968 | 0.140823 | C | -0.84876 | 1.555857 | -0.1059 |
| H | 0.512837 | 0.820775 | 0.169103 | C | -0.99196 | 1.483474 | -0.1113 |
| H | -0.00711 | 1.625319 | -0.55451 | C | -0.94822 | 1.435782 | -0.15718 |
| H | -0.28486 | 1.554506 | -0.48136 | C | -0.76096 | 1.45929 | -0.19818 |
| H | -0.35017 | 1.465326 | -0.41756 | C | -0.60084 | 1.52874 | -0.18992 |
| H | -0.42941 | 1.382223 | -0.33797 | C | -0.64631 | 1.576579 | -0.14452 |
| H | -1.05078 | 1.547587 | -0.28005 | C | -0.61628 | 1.290516 | -0.20468 |
| H | -0.96683 | 1.631975 | -0.35918 | C | -0.7501 | 1.332223 | -0.25 |
| H | -0.98737 | 1.320775 | -0.33091 | C | -0.09178 | -1.17961 | -0.53963 |
| H | -1.49286 | 1.62532 | 0.05452 | C | -0.29685 | -1.18067 | -0.49884 |
| H | -1.21517 | 1.554507 | -0.01863 | C | -0.38823 | -1.109 | -0.47172 |
| H | -1.14996 | 1.465334 | -0.08244 | C | -0.65135 | -1.05586 | -0.3941 |
| H | -1.07079 | 1.382232 | -0.16203 | C | -0.50818 | -0.98347 | -0.3887 |
| H | -0.44938 | 1.54758 | -0.21996 | C | -0.55195 | -0.93578 | -0.34283 |
| H | -0.53327 | 1.631968 | -0.14082 | C | -0.73922 | -0.95929 | -0.30182 |
| H | -0.51284 | 1.320775 | -0.1691 | C | -0.89931 | -1.02874 | -0.31009 |
| H | -0.00711 | -1.12532 | -0.55451 | C | -0.85381 | -1.07658 | -0.35549 |
| H | -0.28486 | -1.05451 | -0.48136 | C | -0.88393 | -0.79052 | -0.29533 |
| H | -0.35017 | -0.96533 | -0.41756 | C | -1.40821 | -1.17961 | 0.039645 |
| H | -0.42941 | -0.88222 | -0.33797 | C | -1.20316 | -1.18067 | -0.00116 |
| H | -1.05078 | -1.04759 | -0.28005 | C | -1.11181 | -1.109 | -0.02828 |
| H | -0.96683 | -1.13197 | -0.35918 | C | -0.84876 | -1.05586 | -0.1059 |
| H | -0.98737 | -0.82077 | -0.33091 | C | -0.99196 | -0.98347 | -0.1113 |
| H | -1.49286 | -1.12532 | 0.05452 | C | -0.94822 | -0.93578 | -0.15718 |
| H | -1.21517 | -1.05451 | -0.01863 | C | -0.76096 | -0.95929 | -0.19818 |
| H | -1.14996 | -0.96533 | -0.08244 | C | -0.60084 | -1.02874 | -0.18992 |
| H | -1.07079 | -0.88223 | -0.16203 | C | -0.64631 | -1.07658 | -0.14452 |
| H | -0.44938 | -1.04758 | -0.21996 | C | -0.61628 | -0.79052 | -0.20468 |
| H | -0.53327 | -1.13197 | -0.14082 | C | -0.7501 | -0.83222 | -0.25 |
| H | -0.51284 | -0.82077 | -0.1691 | C | 0.091782 | -0.67961 | 0.539633 |
| H | 0.007111 | -0.62532 | 0.554506 | C | 0.296848 | -0.68067 | 0.498837 |
| H | 0.284856 | -0.55451 | 0.48136 | C | 0.388227 | -0.609 | 0.471719 |
| H | 0.350168 | -0.46533 | 0.417559 | C | 0.65135 | -0.55586 | 0.394102 |
| H | 0.429412 | -0.38222 | 0.337973 | C | 0.508176 | -0.48347 | 0.3887 |
| H | 1.050775 | -0.54759 | 0.280053 | C | 0.551955 | -0.43578 | 0.34283 |
| H | 0.966827 | -0.63197 | 0.359185 | C | 0.739216 | -0.45929 | 0.301824 |
| H | 0.987366 | -0.32077 | 0.330906 | C | 0.899312 | -0.52874 | 0.310087 |
| H | 1.492864 | -0.62532 | -0.05452 | C | 0.85381 | -0.57658 | 0.355489 |
| H | 1.21517 | -0.55451 | 0.018632 | C | 0.883931 | -0.29052 | 0.295326 |
| H | 1.149965 | -0.46533 | 0.082444 | C | 1.408205 | -0.67961 | -0.03964 |
| H | 1.070788 | -0.38223 | 0.162034 | C | 1.203162 | -0.68067 | 0.001155 |
| H | 0.44938 | -0.54758 | 0.219958 | C | 1.111806 | -0.609 | 0.028276 |
| H | 0.533267 | -0.63197 | 0.140823 | C | 0.848755 | -0.55586 | 0.105902 |
| H | 0.512837 | -0.32077 | 0.169103 | C | 0.991961 | -0.48347 | 0.111304 |
| H | -0.18085 | 0.25 | 0.589233 | C | 0.94822 | -0.43578 | 0.157177 |
| H | 1.680806 | 0.25 | -0.08925 | C | 0.760962 | -0.45929 | 0.198185 |
| H | 0.180848 | 0.75 | -0.58923 | C | 0.60084 | -0.52874 | 0.189922 |
| H | -1.68081 | 0.75 | 0.08925 | C | 0.646305 | -0.57658 | 0.144518 |
| C | 0.091782 | 1.179613 | 0.539633 | C | 0.616278 | -0.29052 | 0.204682 |
| C | 0.296848 | 1.180666 | 0.498837 | C | 0.750105 | -0.33222 | 0.250004 |
| C | 0.388227 | 1.108996 | 0.471719 | C | -0.01204 | 0.25 | 0.559637 |
| C | 0.65135 | 1.055857 | 0.394102 | C | 1.512014 | 0.25 | -0.05965 |
| C | 0.508176 | 0.98347 | 0.3887 | C | 0.012037 | 0.75 | -0.55964 |
| C | 0.551955 | 0.935777 | 0.34283 | C | -1.51201 | 0.75 | 0.05965 |
| C | 0.739216 | 0.959289 | 0.301824 | N | 0.569961 | 1.114522 | 0.432525 |
| C | 0.899312 | 1.028743 | 0.310087 | N | 0.930106 | 1.114522 | 0.067476 |
| C | 0.85381 | 1.076583 | 0.355489 | N | 0.7501 | 0.916379 | 0.250004 |
| C | 0.883931 | 0.790516 | 0.295326 | N | -0.56996 | 1.614522 | -0.43253 |
| C | 1.408205 | 1.179613 | -0.03964 | N | -0.93011 | 1.614522 | -0.06748 |
| C | 1.203162 | 1.180666 | 0.001155 | N | -0.7501 | 1.416379 | -0.25 |
| C | 1.111806 | 1.108996 | 0.028276 | N | -0.56996 | -1.11452 | -0.43253 |
| C | 0.848755 | 1.055857 | 0.105902 | N | -0.93011 | -1.11452 | -0.06748 |
| C | 0.991961 | 0.983474 | 0.111304 | N | -0.7501 | -0.91638 | -0.25 |
| C | 0.94822 | 0.935782 | 0.157177 | N | 0.569961 | -0.61452 | 0.432525 |
| C | 0.760962 | 0.95929 | 0.198185 | N | 0.930106 | -0.61452 | 0.067476 |
| C | 0.60084 | 1.02874 | 0.189922 | N | 0.7501 | -0.41638 | 0.250004 |
| C | 0.646305 | 1.076579 | 0.144518 | N | 0.391131 | 0.25 | 0.479692 |
| C | 0.616278 | 0.790516 | 0.204682 | N | 1.108889 | 0.25 | 0.020303 |
| C | 0.750105 | 0.832223 | 0.250004 | N | -0.39113 | 0.75 | -0.47969 |
| C | -0.09178 | 1.679613 | -0.53963 | N | -1.10889 | 0.75 | -0.0203 |

Table S2. Element distribution of TP-PDA electrode in initial state

| Element | Apparent Concentration | Wt% | Wt% Sigma | Atomic % |
| --- | --- | --- | --- | --- |
| C | 122.79 | 89.97 | 0.84 | 91.78 |
| N | 4.73 | 7.61 | 0.86 | 6.65 |
| F | 4.63 | 2.43 | 0.11 | 1.57 |
| Na | 0.00 | 0.00 | 0.04 | 0.00 |
| P | 0.00 | 0.00 | 0.04 | 0.00 |
| Total: |  | 100.00 |  | 100.00 |

Table S3. Element distribution of TP-PDA electrode after discharge

| Element | Apparent Concentration | Wt% | Wt% Sigma | Atomic % |
| --- | --- | --- | --- | --- |
| C | 80.27 | 77.91 | 0.75 | 84.53 |
| N | 5.86 | 7.13 | 0.88 | 6.64 |
| F | 9.31 | 3.77 | 0.12 | 2.59 |
| Na | 24.18 | 10.57 | 0.14 | 5.99 |
| P | 1.45 | 0.60 | 0.04 | 0.25 |
| Total: |  | 100.00 |  | 100.00 |

Table S4. Element distribution of TP-PDA electrode after charge

| Element | Apparent Concentration | Wt% | Wt% Sigma | Atomic % |
| --- | --- | --- | --- | --- |
| C | 92.91 | 77.53 | 0.39 | 84.02 |
| N | 7.31 | 7.34 | 0.46 | 6.82 |
| F | 24.86 | 8.35 | 0.10 | 5.72 |
| Na | 10.52 | 4.03 | 0.04 | 2.28 |
| P | 8.15 | 2.74 | 0.04 | 1.15 |
| Total: |  | 100.00 |  | 100.00 |

Table S5. Comparison of TP-TA COF with previously reported COFand related polymer-based cathode materials

|  | Battery | Cycle number (n), Current density, Capacity (mAh g^-1^)，Capacity retention rate | Voltage range | Reference |
| --- | --- | --- | --- | --- |
| TP-PDA | SIB | 1800, 3 A g^-1^, 124 mAh g^-1^，97.1% | 1.3 ‒ 4.2 V | This Work |
| S@TAPT-COFs | SIB | 2000, 2 A g^-1^, 68.6 mAh g^-1^，76.0% | 1.5 ‒ 3.2 V | ^[7]^ |
| HATN-PD-COF | SIB | 7000, 10 A g^-1^, 190 mAh g^-1^，91.0% | 1 ‒ 3.6 V | ^[8]^ |
| TPDA-NDI-30%CNT | SIB | 10000, 1 A g^-1^, 49 mAh g^-1^，82.0% | 1 ‒ 3.8V | ^[9]^ |
| TAPB-NDA@50%CNT | SIB | 2000, 0.3 A g^-1^, 80 mAh g^-1^，81.2% | 1.5 ‒ 3.5 V | ^[10]^ |
| TQBQ-COF | SIB | 1000, 1 A g^-1^, 236 mAh g^-1^, 91.3% | 1 ‒ 3.5 V | ^[11]^ |
| IISERP-COF18 | SIB | 1200, 1 A g^-1^, 340 mAh g^-1^，92.0% | 0 ‒ 3 V | ^[12]^ |
| COF_TPDA – PMDA_@50%CNT | LIB | 1800, 5 A g^-1^, 80 mAh g^-1^, 50.0% | 1.2 ‒ 4.3 V | ^[13]^ |
| DAPQ-COF@50%CNT | LIB | 3000, 2 A g^-1^, 119 mAh g^-1^, 76.0% | 1.5 ‒ 3.2 V | ^[14]^ |
| TP-TA COF | LIB | 1500, 5 A g^-1^, 129 mAh g^-1^, 83.3% | 1.2 ‒ 4.3 V | ^[15]^ |
| TpPa-COF@CNT | PIB | 2000, 2 A g^-1^,282.5 mAh g^-1^, 85.6% | 0 ‒ 3 V | ^[16]^ |

**References**

[1] D. Van Der Spoel, E. Lindahl, B. Hess, G. Groenhof, A. E. Mark, H. J. Berendsen, *J. Comput. Chem.* **2005**, *26*, 1701.

[2] J. Wang, R. M. Wolf, J. W. Caldwell, P. A. Kollman, D. A. Case, *J. Comput. Chem.* **2004**, *25*, 1157.

[3] B. Hess, H. Bekker, H. J. Berendsen, J. G. Fraaije, *J. Comput. Chem.* **1997**, *18*, 1463.

[4] T. Darden, D. York, L. Pedersen, *J. Chem. Phys.* **1993**, *98*, 10089.

[5] H. J. Berendsen, J. v. Postma, W. F. Van Gunsteren, A. DiNola, J. R. Haak, *J. Chem. Phys* **1984**, *81*, 3684.

[6] R. Martoňák, A. Laio, M. Parrinello, *Phys. Rev. Lett.* **2003**, *90*, 075503.

[7] J. Shi, W. Tang, B. Xiong, F. Gao, Q. Lu, *Chem. Eng. J.* **2023**, *453*, 139607.

[8] X. Yang, L. Gong, Z. Liu, Q. Zhi, B. Yu, X. Chen, K. Wang, X. Li, D. Qi, J. Jiang, *Sci. China Chem.* **2024**, *67*, 1300.

[9] S. Jindal, Z. Tian, A. Mallick, S. Kandambeth, C. Liu, P. M. Bhatt, X. Zhang, O. Shekhah, H. N. Alshareef, M. Eddaoudi, *Small* **2024**, 2407525.

[10] S. Biswas, A. Pramanik, A. Dey, S. Chattopadhyay, T. S. Pieshkov, S. Bhattacharyya, P. M. Ajayan, T. K. Maji, *Small* **2024**, *20*, 2406173.

[11] R. Shi, L. Liu, Y. Lu, C. Wang, Y. Li, L. Li, Z. Yan, J. Chen, *Nat. Commun.* **2020**, *11*, 178.

[12] S. Haldar, D. Kaleeswaran, D. Rase, K. Roy, S. Ogale, R. Vaidhyanathan, *Nanoscale Horiz.* **2020**, *5*, 1264.

[13] L. Yao, C. Ma, L. Sun, D. Zhang, Y. Chen, E. Jin, X. Song, Z. Liang, K.-X. Wang, *J. Am. Chem. Soc.* **2022**, *144*, 23534.

[14] H. Gao, Q. Zhu, A. R. Neale, M. Bahri, X. Wang, H. Yang, L. Liu, R. Clowes, N. D. Browning, R. S. Sprick, *Adv. Energy Mater.* **2021**, *11*, 2101880.

[15] M. Wu, Y. Zhao, R. Zhao, J. Zhu, J. Liu, Y. Zhang, C. Li, Y. Ma, H. Zhang, Y. Chen, *Adv. Funct. Mater.* **2022**, *32*, 2107703.

[16] D. Yan, L. Song, F. Kang, X. Mo, Y. Lv, J. Sun, H. Tang, X. Zhou, Q. Zhang, *Angew. Chem. Int. Ed.* **2025**, https://doi.org/10.1002/anie.202422851.
